# Tolerance for uncertainty and medical students' specialty choices: A myth revisited

**DOI:** 10.1111/medu.15610

**Published:** 2025-01-23

**Authors:** Odette Wegwarth, Moritz Pfoch, Claudia Spies, Martin Möckel, Stefan J. Schaller, Markus Wehler, Helge Giese

**Affiliations:** ^1^ Heisenberg Chair for Medical Risk Literacy and Evidence‐Based Decisions Charité Universitätsmedizin Berlin Berlin Germany; ^2^ Center for Adaptive Rationality Max Planck Institute for Human Development Berlin Germany; ^3^ Department of Anesthesiology and Intensive Care Medicine (CCM/CVK) Charité — Universitätsmedizin Berlin Berlin Germany; ^4^ Department of Emergency Medicine with Chest Pain Units Charité Universitätsmedizin Berlin Berlin Germany; ^5^ Department of Anesthesia, Intensive Care Medicine and Pain Medicine, Division of General Anesthesia and Intensive Care Medicine Medical University of Vienna Vienna Austria; ^6^ Department of Emergency Medicine Universitätsklinikum Augsburg Augsburg Germany

## Abstract

**Background:**

In 1962, the idea emerged that medical students' tolerance of uncertainty could determine their specialty choice. While some studies supported this claim, others refuted it, often using independently developed instruments. We explored whether the reported link between specialty choice and uncertainty tolerance is more myth than evidence by employing established instruments to investigate whether specialty choice could be explained by variance in uncertainty tolerance.

**Method:**

We conducted a cross‐sectional online survey at two periods of time. From February to June 2023, we queried 563 final‐year medical students from 34 German medical universities (1) on their uncertainty tolerance using three validated tools (the modified tolerance for ambiguity scale, the physicians' reaction to uncertainty scale and the uncertainty intolerance scenario method) and (2) on their intended specialty choice. In a follow‐up 1 year later (May to June 2024), 263 of those medical students responded to our query on their final specialty choice and again on their uncertainty tolerance.

**Results:**

Participants' (*N* = 563) median age was 26.0 years (mean: 27.2; SD = 3.8), and 70% (*n* = 396) were female. Originally reported differences and rank orders in uncertainty tolerance among medical students with different intended specialty choices could not be replicated for any of the three scales. Instead, our results suggest different rank orders of uncertainty tolerance by different tools, as well as nonsignificant differences between intended medical specialties. Intercorrelation coefficient analyses demonstrated that, depending on the scale, only 0.3% to 1.5% of the variance in uncertainty tolerance could be attributed to specialty choice. Follow‐up data using actual instead of intended medical choices left findings unchanged.

**Discussion:**

Our findings suggest that the presumed link between uncertainty tolerance and specialty choice is more myth than evidence. Instead of teaching this link or using it as an admissions criterion, medical schools should equip students with the skills needed to navigate uncertainty across their careers.

## Abstract 2

Does medical students’ uncertainty tolerance really determine their specialty choice? A multi‐center cross‐sectional study from @ChariteBerlin challenges this long‐held belief. #MedEd #SpecialtyChoice

## 1 INTRODUCTION

In 1962, it was suggested that medical students' tolerance for uncertainty—defined as the ability to navigate incomplete, unclear, or unpredictable information and also referred to as *ambiguity*—likely dictates whether they become a psychiatrist or a surgeon. Budner,^1^ a trained personality psychologist, was the first to establish a link between medical students' uncertainty tolerance and their specialty choices. Using a self‐developed scale to measure uncertainty tolerance, he found that students who opted for “softer” specialties, that is nearer to social and humanities science such as psychiatry or internal medicine, exhibited higher tolerance for uncertainty compared to students pursuing “solid” specialties such as surgery. This idea gained traction, particularly among sociologists, psychologists and epidemiologists,^2^, ^3^, ^4^, ^5^ who conducted subsequent studies using modified or new measures. Commonly, their studies echoed Budner's findings, consistently highlighting that individuals aiming to specialise in softer disciplines demonstrated greater tolerance for uncertainty, while those pursuing surgical careers exhibited lower tolerance. However, researchers from fields less tied to the social sciences struggled to replicate the proposed link between uncertainty tolerance and specialty choice,^6^, ^7^, ^8^, ^9^, ^10^ raising concerns about the robustness of earlier studies. Notably, some of the most prominent studies supporting the association between uncertainty tolerance and specialty choice lacked scientific rigour. For instance, in one study, rankings associating psychiatry with the highest uncertainty tolerance were based on subgroups of only six medical students^2^; in another, rankings that initially diverged from Budner's findings were adjusted^4^ to align with prior studies.^1^ In yet another study, uncommon statistical methods, such as comparing median split upper and lower bounds, were employed to establish differences between groups that were not otherwise apparent.^3^ Furthermore, the underlying claim that medical disciplines would inherently differ in their levels of uncertainty has never been systematically investigated or validated. As a result, the proposed link between tolerance for uncertainty and medical students' specialty choices might be more myth than solid evidence.

Despite these challenges, the belief that uncertainty tolerance and medical specialty are linked remains compelling. Recent research that revisited this claim using measures developed by its original proponents^11^ affirmed the once established dualism in uncertainty tolerance between clinical and surgical specialties, with the latter being associated with lower uncertainty tolerance. Uncertainty tolerance has also rather recently been proposed as a criterion for medical school admissions, associated with the claim that it would enhance quality of care in ambiguous situations, address physician supply imbalances, and foster greater humility among physicians.^12^ And lastly and perhaps most concerning, this claim continues to be taught in medical schools.

Given the significant impact this long‐held belief can have on medical school admissions and training, we aimed to revisit the initial claims about the association between specialty choice and uncertainty tolerance, with four specific goals in mind: We sought (1) to replicate the originally reported findings between intended medical specialty choice and uncertainty tolerance using three of the original assessment approaches, (2) to explore how much variance in students' uncertainty tolerance per assessment scale is explained by the specific intended specialty choices, (3) to examine the validity of the scales and (4) to investigate whether results are independent of medical students' intended and actual specialty choices.

## 2 METHOD

The work reported here is a prospective cross‐sectional online survey study. The study design and content were approved by the Institutional Ethics Committee of Charité – Universitätsmedizin Berlin (Germany) (EA4/221/22) and the Medical Intern Committee of Ethics Committee of Charité – Universitätsmedizin Berlin (Germany). Written informed consent was obtained online from all participants at the study's outset. Our reporting follows the Strengthening the Reporting of Observational Studies in Epidemiology (STROBE) guideline (Supporting Information S1).

### 2.1 Participants and recruitment

To obtain insights from members of multiple faculties and to gather information from a broad range of participants who had already been exposed to a variety of medical disciplines, we surveyed medical students in their final practical year (medical interns) from a wide range of medical faculties across Germany. To detect effect sizes as suggested by Geller et al.^2^ (*R* ^2^ = 0.20) and Gerrity et al.^3^ (*R* ^2^ = 0.19) with a power of 0.80 and an alpha of 0.05, we calculated that 200 participants were needed (*f* = 0.24, 1 − β = 0.8, ***α*** = 0.05). To ensure a large and diverse sample, we invited medical intern coordinators from all 40 university hospitals in Germany (37 public and three private) to participate in our study. The announcement, which included a link to the online survey, was distributed via email and on learning platforms for medical interns. This effort ultimately resulted in a sample drawn from interns at 34 medical universities. Supporting information S2 provides details on the participating universities. Altogether, 611 medical students participated between February and June 2023. After removing duplicates (*n* = 31) and those who were not yet in their practical year (*n* = 17), we were left with data from 563 medical interns. The participants who completed the survey and provided their contact information in order to be invited to the follow‐up 1 year later (*n* = 563) were approached again between June and July 2024.

### 2.2 Survey questionnaire

To measure associations between uncertainty tolerance and specialty choice, we selected three validated instruments that reported significant or nonsignificant effects on that phenomenon: Geller et al.'s modified tolerance for ambiguity scale,^2^ Gerrity et al.'s physicians' reaction to uncertainty (PRU) scale^3^—both prominent studies that reported large effects of uncertainty tolerance on specialty choice—and Simpkin et al.'s uncertainty intolerance scenario‐based method,^6^ which reported no effect. Detailed wording for each instrument can be found in Supporting Information S2.

The modified tolerance for ambiguity scale^2^ is an adapted, shorter version of Budner's original instrument.^1^ It consists of four items intended to measure uncertainty tolerance, on a 6‐point rating scale (from ‘I highly disagree’ to ‘I highly agree’). The modified scale introduced an item that was not included in the original version—item 4—that suggested that psychiatrists would be particularly exposed to uncertainty, while surgeons and radiologists would not (‘As a doctor, I would prefer the clear and definitive work of a surgeon or radiologist to the uncertainties of a psychiatrist’), thereby postulating the hypothesis that was yet to be proven. Considering the potential biasing impact of item 4, our study tested whether including or excluding this item affected results.

The PRU scale by Gerrity et al.^3^ is an original scale with 13 items (e.g. ‘the uncertainty of patient care often troubles me’, and ‘I find the uncertainty involved in patient care disconcerting’); the participants rate each item on a 6‐point scale (from ‘I highly disagree’ to ‘I highly agree’). Notably, the initial ranking of uncertainty tolerance per medical specialty choice retrieved by Gerrity et al.^3^ was the reverse of the findings in Geller et al.^2^ and Budner.^1^ Only after adjusting for cofactors like age, gender, and professional experience was the ranking aligned to the earlier findings.

Unlike the other two scales, which treat uncertainty tolerance as a static, situation‐independent trait, Simpkin et al.'s^9^ uncertainty intolerance scenario‐based approach views uncertainty tolerance as situation dependent. This instrument presents four medical scenarios (heart, respiratory system, infectious disease and psychiatric issues), each with four levels of situational uncertainty (from very high to very low), resulting in 16 different scenarios. For example, the heart‐related scenario with the lowest uncertainty reads: ‘Our diagnosis is pericarditis’, while the highest uncertainty scenario read: ‘We suspect pericarditis […] But we are unsure.’ The participants rated their worry about the expressed uncertainty per scenario on a 6‐point scale. The study found that these linguistic variations of uncertainty induced different levels of perceived uncertainty but found no association between these levels and intended medical specialty choice.

The questionnaire for our first study wave included the complete modified tolerance for ambiguity and PRU scales and, for the sake of study parsimony, four scenarios of the uncertainty intolerance scenario‐based instrument, covering each of the four medical conditions with one of the four levels of linguistic uncertainty (very high to very low). We also asked medical students for their intended specialty choice. The follow‐up used the exact same questionnaire as in the first wave but asked medical students now for their actual rather than their intended specialty choice. Detailed wording of the survey can be found in Supporting Information S2.

### 2.3 Primary and secondary endpoint measures

To test the replicability of established orders of uncertainty tolerance per scale and the correlations of orders across scales, the primary endpoint was the mean (possible mean range per scale is 1–6) of uncertainty tolerance per intended specialty choice. While Simpkin et al.^9^ found no association between intended specialty choice and uncertainty tolerance, both Geller et al.^2^ (like Budner^1^) and Gerrity et al.^3^ found considerable differences and reported the lowest tolerance among students intending to become surgeons. They also found higher tolerance among those intending to practice general medicine and internal medicine, with Gerrity et al.^3^ noting that students pursuing internal medicine had higher tolerance than those interested in general medicine. Geller et al.^2^ reported the highest uncertainty tolerance among psychiatry students—a finding based on six participants. This specialty was not evaluated in the other studies because it was not selected by enough students. We tested the descending order of uncertainty tolerance established across these studies, namely, internal medicine > general medicine > surgery.

The secondary endpoint was the proportion of variance of uncertainty tolerance across specialty choices that was explained by specialty choice. The tertiary endpoint was the internal validity of the three assessment instruments used in this study and the Pearson correlation coefficient between them.

### 2.4 Analysis

To (1) replicate the differences in uncertainty tolerance among medical specialty choices across the three scales, we first classified the specialty choices into four groups: surgery/gynaecology (*n* = 136), internal medicine (*n* = 103), primary care (*n* = 50) and other (*n* = 274), including psychiatry (*n* = 34). We then tested Greenhouse–Geisser corrected differences among groups in a 4 (specialty) x 3 (scale) mixed analysis of variance (ANOVA), followed by Bonferroni‐corrected post‐hoc comparisons among groups for significant effects. Eta squared (*η* ^2^) is used to report the magnitude of the effects of the ANOVA, indicating how much of the variance in the dependent variable is accounted for by the independent variable(s).

To (2) explore systematic differences between specialty choices regardless of classification, we separately estimated the intraclass correlation as a measure of variance of uncertainty explained by intended specialty choices for all three scales. To this end, we employed a multilevel linear model with random intercepts and tested the significance with a maximum likelihood *χ* ^2^‐test.

To (3) validate and compare the scales, we assessed internal consistencies and correlations between the scales and estimated the retest reliability with follow‐up data. Furthermore, we used participants' final specialty choices, retrieved during follow‐up, to evaluate the stability of the effects. All effects with at least *P* ≤ 0.05 were deemed significant. Analyses were performed in IBM SPSS 23.0.

## 3 RESULTS

### 3.1 Participants

The median age of participants (*N* = 563) was 26.0 years (mean: 27.2 years, SD = ±3.8). A total of 70.3% (*n* = 396) participants were female, 29.0% (*n* = 163) were male, and 0.4% (*n* = 2) were neither, resulting in slightly more female participants compared to data published by German Medical Association on gender distribution in the general population of medical students, which reports about 67% female students. While we did not apply a quota method, the distribution of the intended medical specialties of our sample was descriptively similar to the specialty distribution in the general population of medical students published by the German Medical Association (Table 1). At follow‐up, 263 participants provided information on their actual specialty choices, with 68.8% (*n* = 181) selecting the same specialty they had intended to choose a year earlier.

**TABLE 1:** Distributions of intended medical specialty between the study sample and a representative population of German medical students as published by the German Medical Association.

Intended medical specialty choice	Sample (%)	Distribution based on a representative survey (%)
Primary care	8.9	11.3
Anaesthesiology	11.0	10.2
Ophthalmology	2.7	1.4
Surgery	11.5	16.1
Gynaecology and obstetrics	11.9	7.5
Ear, nose and throat medicine	1.8	1.4
Dermatology	2.5	1.9
Internal medicine	18.3	18.3
Paediatrics and adolescent medicine	9.8	11.7
Neurology	5.7	5.9
Psychiatry	5.3	2.8
Radiology	3.4	3.0
Urology	3.2	2.1
Other	4.0	6.6

### 3.2 Replicability of rank order

**FIGURE 1:**
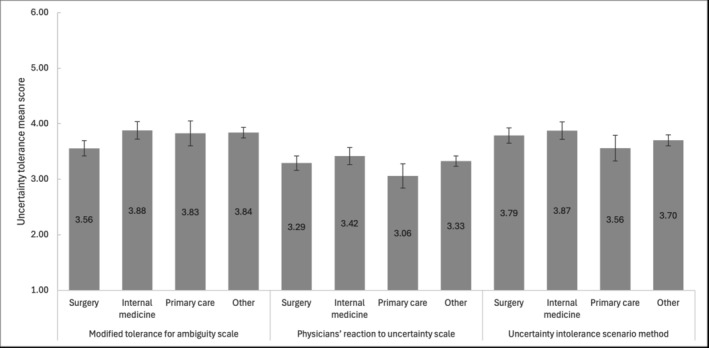
Uncertainty tolerance per specialty and scale. Note. 1: lowest possible uncertainty tolerance; 6: highest possible uncertainty tolerance. Confidence bands show 95% confidence intervals. Values within the bars indicate the mean.

Rank orders of uncertainty tolerance associated with medical specialty choice systematically differed between the three scales (*P* = 0.003, *η* ^2^_*part*_ = 0.02; Figure 1), suggesting a different uncertainty tolerance per medical specialty choice depending on the scale. However, within the scales, rank order in uncertainty tolerance did not differ between specialty choices for either the PRU scale (*P* = 0.069, *η* ^2^_*part*_ = 0.01) or the uncertainty intolerance scenario‐based scale (*P* = 0.11, *η* ^2^_*part*_ = 0.01). We only observed such differences for the modified tolerance for ambiguity scale when including item 4 (*P* = 0.004, *η* ^2^_*part*_ = 0.02). In that case, the participants who selected surgery had lower uncertainty tolerance than did the participants who selected internal medicine and other specialties. When excluding item 4, however, no systematic differences (*P* = 0.41, *η* ^2^_*part*_ = 0.01) between specialty choices were observed for that scale either. These patterns remained unchanged for actual specialty choices at follow‐up.

### 3.3 Variance in uncertainty tolerance explained by specialty per scale

**FIGURE 2:**
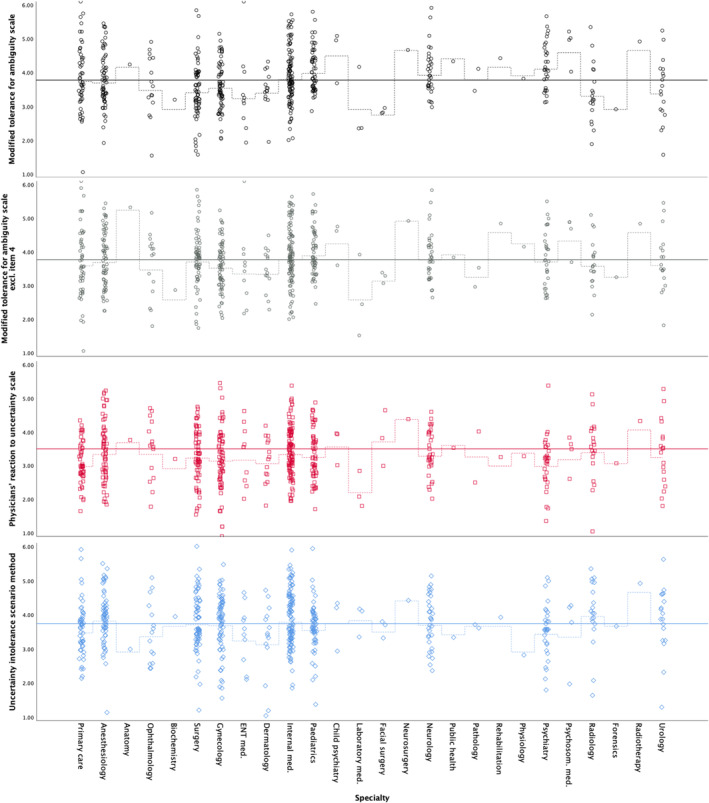
Individual distribution of uncertainty tolerance across each medical specialty per scale. *Note.* Dotted lines depict the mean of each specialty; a jitter is applied to enhance legibility. 1: lowest possible uncertainty tolerance; 6: highest possible uncertainty tolerance. [Color figure can be viewed at wileyonlinelibrary.com]

No variance in uncertainty tolerance could be sufficiently explained by the participants' intended specialty choice in any of the three assessment instruments (all below 1.5%; *χ* ^2^(1) ≤ 1.52, *P* ≥ 0.22; Figure 2). The intraclass correlation coefficient ranged between 0.005 and 0.003 and did not reach the significance level for any uncertainty assessment instruments (Table 2). A weak association was established in the modified tolerance for ambiguity scale only when item 4 (which explicitly mentioned psychiatry and surgery) was included, explaining approximately 8% of the variance in uncertainty tolerance (*χ* ^2^(1) ≤ 15.09, *P* ≤ 0.001; Table 2). Tested on its own, item 4 explained a full 26.6% of the variance (*χ* ^2^(1) = 87.60, *P* ≤ 0.001), highlighting its highly suggestive effect. These patterns remained unchanged when analysing actual specialty choices at follow‐up (Table 2).

**TABLE 2:** Variance of uncertainty tolerance explained by medical choices, measured by intraclass correlation.

Scale	Intraclass correlation: First wave (variance in percent)	Intraclass correlation: Follow‐up (variance in percent)
Modified tolerance for ambiguity scale	0.081* (8.1%)	0.105* (10.5%)
Modified tolerance for ambiguity scale, excl. item 4	0.003 (0.3%)	0.007 (0.7%)
Modified tolerance for ambiguity scale, item 4 only	0.266* (26.6%)	0.263* (26.3%)
Physicians' reaction to uncertainty scale	0.005 (0.5%)	0.007 (0.7%)
Uncertainty intolerance scenario method	0.015 (1.5%)	0.001 (0.1%)

*Note*: *P < 0.05.

### 3.4 Validation of established scales

Given its 13 items, the PRU scale^3^ had the highest internal validity (*α* = 0.89), followed by the scenario‐based approach,^9^ which reached an internal validity of *α* = 0.79 with just the four scenarios we used in our study (instead of the 16 scenarios in the original version). Both scales also showed the highest correlation (Table 3) with each other. The internal consistency of the modified tolerance for ambiguity scale,^2^ including item 4, was poor (*α* = 0.58). Notably, the reliability and validity of this scale improved when the problematic item 4 was excluded. This supports the idea that defining uncertainty tolerance through suggestive item wording is not a valid assessment approach.

**TABLE 3:** Validity of the scales.

Scale	Internal consistency (α)	Retest reliability (r_tt_)	Modified tolerance for ambiguity scale (1)	Modified tolerance for ambiguity scale excl. item 4 (2)	Physicians' reaction to uncertainty scale (3)	Uncertainty intolerance scenario method (4)
Modified tolerance for ambiguity scale (1)	0.58	0.63	‐‐	0.89*	0.24*	0.21*
Modified tolerance for ambiguity scale excl. item 4 (2)	0.61	0.61	0.89*	‐‐	0.31*	0.27*
Physicians' reaction to uncertainty scale (3)	0.89	0.66	0.24*	0.31*	‐‐	0.56*
Uncertainty intolerance scenario method (4)	0.79	0.46	0.21*	0.27*	0.56*	‐‐

*Note*: Pearson correlations are reported unless otherwise specified.

* *P* < 0.05.

## 4 DISCUSSION

Our study aimed to systematically revisit the longstanding claim that uncertainty tolerance influences medical specialty choice, which is at the heart of rather recent calls^12^ to make uncertainty tolerance a criterion in medical school admission processes. For some of the most prominent assessment instruments,^2^, ^3^, ^4^ which continue to influence research today,^11^ we were unable to validate the originally postulated rank orders of uncertainty tolerance or find any significant differences in specialty choice across the scales. Furthermore, we did not observe that variance in uncertainty tolerance, as measured by these instruments, could be explained by specialty choice, further arguing against the existence of a systematic relationship between the two. Notably, the correlation between the three assessment instruments—each purportedly designed to measure the same phenomenon, uncertainty tolerance—was generally low, suggesting that these scales measure distinct constructs rather than a unified concept.

While we are not aware of any other studies that used a comprehensive battery of established tools or that systematically examined the variance in uncertainty tolerance explained by specialty choice, we are not the first to fail to establish a link between students' tolerance for uncertainty and their specialty choice.^7^, ^9^, ^10^ Nevertheless, studies revisiting and promoting a connection between uncertainty tolerance and medical specialty continue to appear, calls to incorporate uncertainty tolerance as a criterion for medical school admissions are published in accredited journals, and, perhaps most concerningly, the concept is still being taught in medical schools. This raises a compelling question: What makes this idea—or, more appropriately given the lack of empirical evidence, this myth—so enduringly appealing?

Myths can serve different social functions.^13^ First, the myth of an association between medical students' uncertainty tolerance and their specialty choice resonates with a broader myth that is deeply ingrained in medical education and historically tied to personality testing: the myth of the ideal candidate. Medical schools face the challenge of selecting applicants who will not only excel academically but also demonstrate characteristics that are essential for a career in medicine, such as compassion, teamwork and integrity. The acknowledgement that being a capable medical student or doctor requires more than academic performance aligns with a wider body of research in general education, which suggests that various noncognitive skills are associated with positive academic and work‐related outcomes in young people.^14^ In light of these findings, the assessment of non‐academic factors, including personality traits, has become more prominent in medical school selection.^15^ However, personality assessments often fail to reliably predict medical school performance or future clinical competence,^16^ which makes their effectiveness questionable.^17^ Personality testing has also raised ethical concerns about fairness and the potential for bias, further complicating their role in medical school admissions.^18^

What personality testing and assessments for uncertainty tolerance have in common is the underlying assumption that human behaviour and characteristics are stable across contexts and time. However, studies demonstrate that this underlying assumption is incorrect: Neither personality traits nor tolerance for uncertainty are stable across contexts and time. Furthermore, both can be effectively influenced by medical education. For instance, a study assessing medical students' personality traits at the beginning and the end of their 6‐year training found significant changes in neuroticism, agreeableness, and conscientiousness, suggesting that medical education influences personality development.^19^ Likewise, a scoping review that examined interventions aimed at improving uncertainty tolerance in medical students concluded that targeted educational strategies could enhance students' ability to manage uncertainty.^20^

Second, the myth of an association between uncertainty tolerance and medical specialty also serves an important social function by providing a practical tool—or, more accurately, a cognitive shortcut—that simplifies the complexities of decision‐making. The availability of seemingly straightforward instruments, such as the Big Five Inventory or the PRU scale, offers an appealing way to distil the multifaceted process of evaluating a good candidate into a single, quantifiable score. This not only alleviates the cognitive burden associated with nuanced decision‐making but also enables decision‐makers to defer accountability to apparently objective metrics—even if they ultimately cannot capture the intricacies of human behaviour or potential. There is a clear tension between the desire for efficiency and the need for meaningful, individualised assessment.

Third, the myth of an association between medical students' uncertainty tolerance and specialty choice may serve to establish or maintain social power. The earliest and most prominent findings suggesting that students favouring ‘softer’ disciplines such as psychiatry were also the most tolerant of uncertainty were primarily advanced by researchers from these very disciplines—psychologists, sociologists and epidemiologists. In the field of medicine, which has traditionally prioritised a biomedical orientation over knowledge rooted in the social sciences or humanities,^13^ this narrative may unintentionally promote a self‐reinforcing perception that students inclined toward these ‘softer’ specialties are in fact superior to their less uncertainty‐tolerant peers, making the idea particularly compelling.

And finally, the myth also aligns well with longstanding stereotypes that characterise certain specialties, such as surgery, as being less reflective or introspective compared to others, such as internal medicine or psychiatry. These stereotypes, often reinforced through jokes portraying surgeons as overly mechanical and clinicians as thoughtful and analytical, provide a convenient framework for categorising complex professional identities, which likely contributes to the enduring appeal of the myth. By simplifying the diverse skillsets required across specialties, these stereotypes offer a seemingly logical but ultimately reductive explanation for professional choices, making the myth difficult to dispel.

While our study suggests that uncertainty tolerance—as measured and constructed by current scales—plays no major role in determining medical students' specialty choice, this does not diminish the fact that uncertainty is a fundamental aspect of all medical practice. Our findings emphasise that uncertainty affects every specialty. Physicians across all disciplines must navigate unpredictable patient outcomes, incomplete information and complex decision‐making. Suggesting that levels of uncertainty vary significantly between specialties not only fails to acknowledge that uncertainty is a universal feature of medicine, it also does a disservice to both medical education and the profession's broader understanding of itself. A recent systematic review has shown that medical education interventions such as interactive acting workshops, simulated clinical sessions and clinical reflection training positively impact medical students' tolerance of uncertainty.^20^ These and other educational interventions help prepare medical students to navigate the uncertainty inherent in any specialty.

This leaves the question of what factors actually influence specialty choice. Numerous studies have been published on this topic, many of which are based on single‐centre and relatively small samples, making them potentially subject to the same limitations identified in the fields of personality trait and uncertainty tolerance testing. A recent systematic review^21^ of 75 studies published between 1977 and 2018, encompassing data from 882 209 individuals, found that the factors influencing medical students' choice of specialty primarily include academic interests (75.3%), competences (55.2%), controllable lifestyles or flexible work schedules (53.0%), patient service orientation (50.0%), medical teachers or mentors (46.9%), career opportunities (44.0%), workload or working hours (37.9%), income (34.7%), length of training (32.3%), prestige (31.17%), advice from others (28.24%) and student debt (15.33%)—although there was significant heterogeneity across studies. Neither personality traits nor uncertainty tolerance were the main factors in medical specialty choice.

### 4.1 Strengths and limitations

Our study has both strengths and limitations. A notable strength of our study is its large, nationwide sample comprising students from medical universities across Germany. This diversity enhances the robustness of our findings and helps mitigate the influence of differing teaching styles across institutions. We also employed three established tools rather than developing our own measures, creating a more robust replication environment and increasing the reliability of our results. Furthermore, our longitudinal study design not only allowed us to associate actual, rather than intended, specialty choice with uncertainty tolerance, but also provided additional validation for the stability of our results. However, one limitation of our study is the generalisability of our findings, as the sample was limited to medical students in Germany. Specialty choice in Germany may occur at a different stage of the postgraduate program compared to other countries, which could affect the applicability of our results internationally. Generalisability may also be constrained by the possibility of nonresponse bias. Nevertheless, for the most critical variable of interest—students' intended specialty choice—we achieved specialty proportions comparable to those reported in representative surveys.

## 5 CONCLUSION

Our study found no evidence to support the longstanding myth that tolerance for uncertainty influences medical specialty choice. A multifaceted approach is needed to effectively combat this potentially harmful myth. First, medical education must emphasise the universal importance of uncertainty tolerance across all specialties, framing it as a skill that evolves with training rather than as a fixed trait. Second, admissions processes should prioritise holistic and evidence‐based evaluations, focussing on candidates' adaptability and potential for growth rather than relying on overly simplistic metrics. Third, medical educators and researchers should actively challenge stereotypes that perpetuate the myth by promoting nuanced discussions about the diverse skillsets required in all specialties. Finally, fostering critical reflection within the medical community about the social functions of myths and their potential biases can help dispel outdated narratives and encourage more equitable practices in medical education.

## AUTHOR CONTRIBUTIONS

**Odette Wegwarth:** Conceptualization; investigation; funding acquisition; writing—original draft; methodology; validation; writing—review and editing; project administration; supervision; resources; formal analysis; visualization; software; data curation. **Moritz Pfoch:** Investigation; data curation; project administration; formal analysis; validation; software; methodology. **Claudia Spies:** Writing—review and editing; supervision; resources; methodology. **Martin Möckel:** Writing—review and editing; conceptualization; methodology. **Stefan J. Schaller:** Writing—review and editing; methodology; supervision. **Markus Wehler:** Methodology; writing—review and editing; conceptualization. **Helge Giese:** Investigation; writing—review and editing; visualization; validation; methodology; software; formal analysis; supervision; data curation; project administration.

## ETHICS STATEMENT

The study design and content were approved by the Institutional Ethics Committee of Charité – Universitätsmedizin Berlin (Germany) (EA4/221/22).

## Supporting information


**Data S1.** Supporting Information.

## Data Availability

Deidentified data and R code will be made available upon publication on OSF (https://osf.io/d4mk8/).
