# Supplementary material for: Tolerance for uncertainty and medical students' specialty choices: A myth revisited
Source: Med Educ. 2025 Jan 23;59(8):833–41. doi: 10.1111/medu.15610 (PMC12242894; doi:10.1111/medu.15610)
Supplement: Supplementary file 1 — Data S1. Supporting Information. [file MEDU-59-833-s001.docx]

**Supplement 2.**

The authors, **Tolerance for uncertainty and medical students’ specialty choices: A theory revisited.**

Content

1. Participating Universities – number participants blinded
2. Survey Items in the Order of Appearance – Please note: All survey items were translated into German

**Participating Universities**

| **Medical University** | **Number of Participants** |
| --- | --- |
| Aachen | xx |
| Augsburg | xx |
| Berlin | xx |
| Bochum | xx |
| Bonn | xx |
| Brandenburg | xx |
| Dresden | xx |
| Düsseldorf | xx |
| Erlangen-Nürnberg | xx |
| Duisburg-Essen | xx |
| Frankfurt am Main | xx |
| Freiburg | xx |
| Gießen | xx |
| Göttingen | xx |
| Greifswald | xx |
| Halle-Wittenberg | xx |
| Hamburg | xx |
| Hannover | xx |
| Heidelberg | xx |
| Homburg | xx |
| Jena | xx |
| Kiel | xx |
| Köln | xx |
| Leipzig | xx |
| Mainz | xx |
| Marburg | xx |
| München (LMU) | xx |
| München (TU) | xx |
| Münster | xx |
| Regensburg | xx |
| Rostock | xx |
| Tübingen | xx |
| Ulm | xx |
| Witten-Herdecke | xx |

**Survey questionnaire**

| **Demographical questions/ specialty choice** |
| --- |
| D1. How old are you? ____ |
| D2. With which gender do you identify with?  *Male, female, diverse* |
| D3. Which specialist medical training would you most like to do at the moment?  *General medicine, Anesthesiology, Anatomy, Ophthalmology, Biochemistry, Surgery, Gynecology, ENT med., Dermatology, Internal medicine, Pediatrics, Child psychiatry, Laboratory medicine, Facial surgery, Neurosurgery, Neurology, Public health, Pathology, Rehabilitation, Physiology, Psychiatry, Psychosomatic medicine, Radiology, Forensics, Radiotherapy, Urology* |
| D4. Do you currently plan to do the additional training as emergency doctor?  *Yes, No, Probably* |
| D5. Have you ever worked full-time?  *Yes, No* |
| D5.1 IF YES: For how many years did you work full time? ______ |
| D6. Where did you study medicine?  *Aachen, Augsburg, Berlin, Bochum, Bonn, Brandenburg, Dresden, Düsseldorf, Erlangen-Nürnberg, Duisburg-Essen, Frankfurt (Main), Freiburg, Gießen, Göttingen, Greifswald, Halle-Wittenberg, Hamburg, Hannover, Heidelberg, Homburg, Jena, Kiel, Köln, Leipzig, Lübeck, Magdeburg, Mainz, Marburg, München (LMU), München (TU), Münster, Oldenburg, Potsdam, Regensburg, Rostock, Tübingen, Ulm, Witten-Herdecke, Würzburg* |
| **Stress from Uncertainty Scale** (Gerrity, Earp, DeVellis, & Light, 1992)  *Strongly disagree (1); Moderately disagree (2); Slightly disagree (3); Slightly agree (4); Moderately agree (5); Strongly agree (6)* |
| ***SUS1.*** The uncertainty of patient care often troubles me. |
| ***SUS2.*** Not being sure of what is best for a patient is one of the most stressful parts of being a physician. |
| ***SUS3.*** I am tolerant of the uncertainties present in patient care. |
| ***SUS4.*** I find the uncertainty involved in patient care disconcerting. |
| ***SUS5.*** I usually feel anxious when I am not sure of a diagnosis. |
| ***SUS6.*** When I am uncertain of a diagnosis, I imagine all sorts of bad scenarios-patient dies, patient sues, etc. |
| ***SUS7.*** I am frustrated when I do not know a patient's diagnosis. |
| ***SUS8.*** I fear being held accountable for the limits of my knowledge. |
| ***SUS9.*** Uncertainty in patient care makes me uneasy. |
| ***SUS10.*** I worry about malpractice when I do not know a patient's diagnosis |
| ***SUS11.*** The vastness of the information physicians are expected to know overwhelms me. |
| ***SUS12.*** I frequently wish I had gone into a specialty or subspecialty that would minimize the uncertainties of patient care. |
| **SUS13.** I am quite comfortable with the uncertainty in patient care. |
| **Situational Approach** (Simpkin, Murphy & Armstrong, 2019)  *Strongly disagree (1); Moderately disagree (2); Slightly disagree (3); Slightly agree (4); Moderately agree (5); Strongly agree (6)* |
| ***Q1.*** I am confident that I know what is going on with this patient*.* |
| ***Q2.*** In a real admission scenario, I would feel the potential uncertainty of the diagnosis of the described patient as discomforting. |
| **Situation 1.** You are sitting in the call room when you receive a page from the emergency room resident: Hey, we have a 55-year-old male who’s presented with central chest pain which is worse on breathing in. He was at a party last night and vomited a few times. He’s had some flu-like symptoms over the past week and has taken quite a lot of Tylenol and Advil. He has no past medical history and a normal examination. His EKG shows normal sinus rhythm, with non-specific ST changes in some of the leads. His troponin is normal. Our working diagnosis is pericarditis and we’ve started high dose NSAIDs and colchicine for the pain. We are also considering whether something else could be going on, such as unstable angina, esophagitis, or coronary spasm due to cocaine taken at the party. |
| **Situation 2.** You are sitting in the call room when you receive a page from the emergency room resident: Hey, we have a 35-year-old woman who’s been brought into ED in an agitated and aggressive state. His mother is with him and says he has a history of amphetamine misuse. She says he had flu-like symptoms for a week and woke up this morning feeling unwell with generalized body pain. He has a GCS of 11 (M5, E4, V2). We haven’t been able to perform a full physical examination due to his agitation. We’ve sent routine bloods and toxicology screen. CT head was negative. Our hypothesis is amphetamine overdose and we’ve started her on IV diazepam. |
| **Situation 3.** You are sitting in the call room when you receive a page from the emergency room resident: Hey, we have a 50-year-old guy who’s come in with a 3-day history of lobe pneumonia worsening cough and shortness of breath. He’s got known COPD and a 30-pack year history of smoking. He’s on amlodipine. He has crackles at the right base and a CXR shows bilateral hazy opacification, worse on the right. Our diagnosis is right lower lobe pneumonia and we’ve started him on antibiotics. |
| **Situation 4.** You are sitting in the call room when you receive a page from the emergency room resident: Hey, we have a 60-year-old female who has presented with flank pain. She reports feeling ill for a couple of days. She is tachycardic with a heart rate of 100 and has leukocyte-positive urine. She looks a little dehydrated with reduced skin turgor. Our working diagnosis is pyelonephritis and we’ve started her on IV antibiotics. We are also considering whether something else could be going on, such as gastroenteritis, diverticulitis, or inflammatory bowel disease. |
| **Tolerance for Ambiguity** (Geller, Faden, & Levine, 1990)  *Strongly disagree (1); Moderately disagree (2); Slightly disagree (3); Slightly agree (4); Moderately agree (5); Strongly agree (6)* |
| ***TA1.*** A good job is one where what is to be done and how it is to be done are always clear. |
| ***TA2.*** In medicine as in other professions, it is possible to get more done by tackling small, simple problems rather than large and complicated ones. |
| ***TA3.*** What we are used to is always preferable to what is unfamiliar. |
| ***TA4.*** As a doctor I would prefer the clear and definite work of someone like a surgeon or radiologist to the uncertainties of a psychiatrist. |

**Follow-up question**

| **Demographical questions/ specialty choice** |
| --- |
| FU1. Which specialist medical training have you eventually started, or are about to start?  *General medicine, Anesthesiology, Anatomy, Ophthalmology, Biochemistry, Surgery, Gynecology, ENT med., Dermatology, Internal medicine, Pediatrics, Child psychiatry, Laboratory medicine, Facial surgery, Neurosurgery, Neurology, Public health, Pathology, Rehabilitation, Physiology, Psychiatry, Psychosomatic medicine, Radiology, Forensics, Radiotherapy, Urology* |
